# Defining Models to Classify between Benign and Malignant Adnexal Masses Using Routine Laboratory Parameters

**DOI:** 10.3390/cancers14133210

**Published:** 2022-06-30

**Authors:** Elisabeth Reiser, Dietmar Pils, Christoph Grimm, Ines Hoffmann, Stephan Polterauer, Marlene Kranawetter, Stefanie Aust

**Affiliations:** 1 Department of Gynecological Endocrinology and Reproductive Medicine, Medical University of Innsbruck, 6020 Innsbruck, Austria; 2 Division of Visceral Surgery, Department of General Surgery, Comprehensive Cancer Center (CCC), Medical University of Vienna, 1090 Vienna, Austria; dietmar.pils@meduniwien.ac.at; 3 Department of Gynecology and Gynecologic Oncology, Comprehensive Cancer Center (CCC), Medical University of Vienna, 1090 Vienna, Austria; ines.hoffmann@bbeisen.at (I.H.); stephan.polterauer@meduniwien.ac.at (S.P.); marlene.kranawetter@meduniwien.ac.at (M.K.); stefanie.aust@meduniwien.ac.at (S.A.)

**Keywords:** adnexal mass, ovarian cancer, benign cyst, BTO, classification, model

## Abstract

**Simple Summary:**

In patients with adnexal masses, classification into benign or malignant tumors is essential for optimal treatment planning, but remains challenging. In the search for new models applicable in a routine clinical setting, we compared classical single parameters to multiparameter predictive models.

**Abstract:**

Discrimination between benign and malignant adnexal masses is essential for optimal treatment planning, but still remains challenging in a routine clinical setting. In this retrospective study, we aimed to compare albumin as a single parameter to calculate models by analyzing laboratory parameters of 1552 patients with an adnexal mass (epithelial ovarian cancer (EOC): *n*= 294; borderline tumor of the ovary (BTO): *n* = 66; benign adnexal mass: *n* = 1192) undergoing surgery. Models comprising classical laboratory parameters show better accuracies (AUCs 0.92–0.93; 95% CI 0.90–0.95) compared to the use of single markers, and could easily be implemented in clinical practice by containing only readily available markers. This has been incorporated into a nomogram.

## 1. Introduction

Epithelial ovarian cancer (EOC) is the most lethal gynecologic malignancy and the fifth most frequent cause of cancer-related mortality in women. The majority of women undergoing surgery for an adnexal mass are diagnosed with a benign histology. In patients with adnexal masses, classification into benign, malignant, or borderline tumors is essential for optimal treatment planning [1]. In a routine clinical setting, primarily transvaginal ultrasound is used to further characterize adnexal masses. Even though specific ultrasound criteria help to improve the predictive accuracy [2], the precise preoperative classification remains challenging, particularly if expertise in ultrasonography is lacking. Thus, treatment and particularly surgery planning should always be based on a combination of factors, including clinical and radiological findings as well as laboratory markers [3].

Cancer antigen 125 (CA125) remains the most used laboratory marker in the diagnostic workup of suspicious adnexal masses [4,5]. Still, the accuracy of this biomarker is limited, as elevated levels of CA125 can also be associated with a variety of benign conditions [6], particularly in premenopausal women and it underperforms in early-stage disease [7], as well as in endometrioid or clear cell histology [8]. 

In the search for other predictive serum markers, preoperative hypoalbuminemia was described as a prognostic parameter in ovarian cancer [9] and other gynecologic malignancies [10,11]. Likewise, C-reactive protein (CRP), gamma glutamyl transferase (GGT), and lactate dehydrogenase (LDH) were identified to be promising in the differential diagnosis of adnexal masses [12,13,14], whereby CRP seems to have a stronger predictive potential with a sensitivity and specificity of 80.1% and 90.8%, respectively, in combination with CA125, whereas GGT showed a sensitivity and specificity of 54.5% and 83.8%. A variety of biomarker panels has been proposed, but none have been comprehensively used in clinical routine thus far.

The aim of the study was, thus, to evaluate the strength of the single parameters albumin, CRP, CA125, and LDH in comparison to models estimated from easily accessible parameters used in clinical routine to differentiate between benign and nonbenign adnexal masses. We performed a relaxed lasso model building procedure using standard laboratory data derived from a routine clinical setting (albumin, erythrocyte, platelet and leucocyte count, hemoglobin, CRP, GGT, alkaline phosphatase (AP), LDH, and fibrinogen), common tumor markers (CA125, cancer Antigen 19-9 (CA19-9), and carcinoembryonic antigen (CEA)) and patients’ age at diagnosis. 

## 2. Materials and Methods

### 2.1. Patients

In this retrospective study, all patients who underwent surgery for adnexal masses (epithelial ovarian cancer (EOC), borderline tumor of the ovary (BTO), and benign adnexal masses) were screened. Thereof, all patients with pretherapeutic available albumin serum levels, adequate preoperative diagnostic workup, and final histologic report were included in the study. The study was approved by the institutional review board (1062/2015 Medical University of Vienna). All patients underwent surgery at the Department of General Gynecology and Gynecologic Oncology, Gynecologic Cancer Unit, Comprehensive Cancer Center, Medical University of Vienna, Austria, between January 2000 and December 2012.

Pretherapeutically, the following measurements were performed: blood tests and physical examination as well as transvaginal ultrasound by an expert sonographer specialized in gynecologic ultrasound. In the case of a suspicious process, further imaging, such as computer tomography or magnetic resonance imaging, was initiated. 

Surgery included cystectomy or unilateral/bilateral salpingo-oophorectomy according to standards of our institution. Patients with EOC or BTO received adequate staging surgery according to AGO guidelines. 

In all cases, pathology assessment was performed by a pathologist specialized in gynecologic oncology. The cases with an inexplicit result were discussed and presented to a second expert pathologist for final pathologic results.

The following parameters were obtained in preoperative blood tests at our department: erythrocyte, platelet and leucocyte count, hemoglobin, albumin, CRP, GGT, AP, LDH, and fibrinogen, as well as the markers CA 19-9, CEA, and CA125. Not all values were available in all patients; thus, missing values were imputed as described in the methods section. 

### 2.2. Statistics

**Figure 1:**
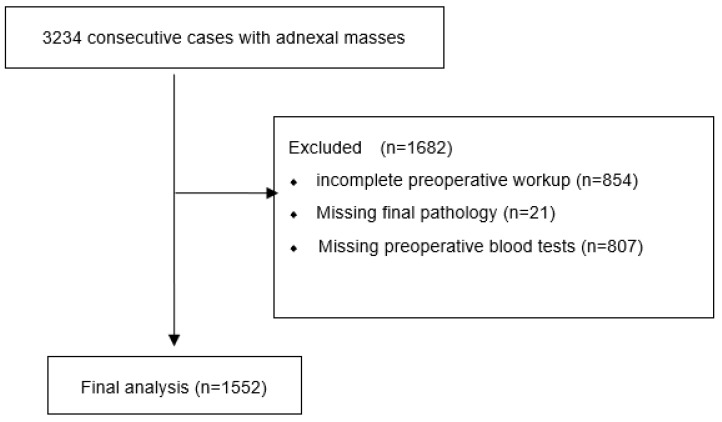
CONSORT diagram describing the identification process and reasons for exclusion of the present retrospective analysis.

All datasets with present albumin values (*n* = 1552) were selected in all three groups of patients, i.e., patients with benign (*n* = 1192), borderline (*n* = 66), or malignant disease (*n* = 294) (Figure 1). The following parameters were considered: age at diagnosis, albumin, CRP, GGT, AP, LDH, erythrocytes, hemoglobin, thrombocytes, leucocytes, fibrinogen, CA125, CA199, and CEA. Missing values are shown in Supplementary Figure S1, plotted with the vis_miss function from R-package naniar 0.6.1. The character of missing values (missing completely at random (MCAR) or not) was tested for each group with the mcar_test (naniar). Missing data were MCAR in the groups borderline (*p* = 0.857) and malignant disease (*p* = 0.883), but not in the benign group (*p* < 0.001). In this group, there were many significant correlations between missingness for nearly all parameters, but missingness of the four most relevant parameters (i.e., age and albumin, as they were complete, and CA125 with LDH) was not correlated. For detailed analyses of correlations of missingness, see file “Missing.txt” in the zip-file of the R-script). There were multiple missing values (*n* = 20) imputed in each group (benign, BOT, and EOC) using function mice of R-package mice 3.14.0 (method = “pmm”; maxit = 50) using all variables to inform the imputation, including histology (which was not used for model building). For model building, all values were log_2_ transformed (offset 0.01 for zero values) to obtain as good as possible pseudonormal distributions (Q–Q plots are shown for all groups and variables in Supplementary Figure S2). All following analyses and model building processes were performed with the complete dataset (*n* = 525 in total, 342 benign, 31 BOT, and 152 EOC) and the 20 imputed datasets and results of all 21 datasets were presented. For all analyses, both nonbenign samples, borderline (BTO), and malignant (*n* = 360) were put together and compared to all benign samples (*n* = 1192). 

Aiming for as sparse as possible models, the relaxed lasso method as implemented in the R-package glmnet 4.1-4 was employed (relax = TRUE; family = “binomial”) [15] on log_2_ transformed (offset 0.01 for zero values) data. To tune the lambda and gamma parameter, 10-fold cross-validation was performed, and for each imputed dataset, the lambda.1se and the gamma.1se values (each the largest value, such that error was within one standard error of the minimum) were used for building of the final model. Four parameters were always selected by the relaxed lasso procedure with all 20 imputed datasets and the complete dataset, i.e., age, LDH, CA125, and albumin. CRP was selected in 13 models due to being known to be a good diagnostic marker for malignancy, and was, therefore, also analyzed in more detail. Parameters CEA (15×), thrombocytes (1×), and AP (1×) were also selected for some models. 

To characterize the predictive values and qualities of the four single parameters (Albumin, CA125, CRP, and LDH) and the corresponding relaxed lasso models (comprised of four to seven parameters), sensitivities, specificities, and accuracies after optimal cutoff definition using the cutpointr function of R-package cutpointr 1.1.2 maximizing the sum of the sensitivity and the specificity (method = maximize_metric; metric = sum_sens_spec) and the area under the receiver operating characteristic (ROC) curves (AUC) with confidence intervals were plotted for each dataset. To compare the AUC of CA125 (the best single diagnostic marker) with the corresponding lasso model, the function roc.test from R-package pROC 1.18.0 (method = “bootstrap”; boot.*n* = 10,000; boot.stratified = TRUE; ties.method = “first”; conf.level = 0.95) was used. Confidence intervals for AUCs were computed with the ci.auc function from R-package pROC using 2000 stratified bootstrap replicates. Additionally, to assess the accuracy of the predictions of the single parameters and the lasso models, the Brier score was calculated from all 20 imputations and the complete dataset was shown as box plots. Calibration curves of all 20 lasso models were estimated and revealed to look very similar. In Supplementary Figure S3, two of them are shown (each plotted using the functions val.prob from R-package rms 6.3-0 (left plot) and val.prob.ci.2 from R-package CalibrationCurves 0.1.2 (right plot), one from a model with four parameters (the smallest ones) and one from a model with seven parameters (the largest ones). All models seemed to underestimate the risk for malignancy a little bit, the larger model less so compared with the smaller model. Finally, to compare specificities of CA125, the best diagnostic single parameter and the lasso models at set sensitivities of 90%, 95%, and 99% are shown. The R-script and the raw data were provided in the Supplementary Materials.

A final logistic regression model was built from a combined dataset (all data from the 20 imputed samples were averaged) employing the four parameters always called age, albumin, CA125, and LDH. This model was provided as an interactive nomogram (built with R-package DynNom 5.0.1) and hosted at the https://www.shinyapps.io/ homepage (accessed on 21 June 2022) under the link https://pils.shinyapps.io/AROMA/ (accessed on 21 June 2022) (using the R-package rsconnect 0.8.26).

## 3. Results

### 3.1. Patients’ Characteristics 

Of the total 1552 included cases, 294 (18.9%) patients were diagnosed with EOC, 66 (4.2%) with BTO, and 1192 (76.8%) had a benign adnexal mass. Within the EOC group, the histologic subtype was as follows: 176 (59.8%) high-grade serous, 14 (4.8%) low-grade serous, 38 (12.9%) high-grade endometrioid, 16 (5.4%) low-grade endometrioid, 15 (5.1%) mucinous, 9 (3.1%) clear cell, and 20 (6.8%) others. FIGO stage I, II, III, and IV accounted for 46 (15.6%), 36 (12.2%), 173 (58.8%), and 39 (13.3%) cases, respectively. 

Within the group of benign adnexal masses, the most common final histologic results included endometrioma (*n* = 254; 21.3%), differentiated teratoma (*n* = 166; 13.9%), simple benign cyst (*n* = 173; 14.5%), serous cystadenoma (*n* = 170; 14.3%), and mucinous cystadenoma (*n* = 136; 11.4%), followed by functional ovarian cyst (*n* = 102; 8.6%), sacto/hydrosalpinx (*n* = 75; 6.3%); ovarian fibroma (*n* = 71; 6.0%), pseudocyst (*n*= 17; 1.4%), hydatid cyst (*n* = 12; 1.0%), benign Brenner tumor (*n* = 7; 0.6%), and other benign ovarian masses (*n*= 9; 0.7%). 

Patients’ characteristics dependent on histologic type are shown in Table 1. 

### 3.2. Correlation

**Figure 2:**
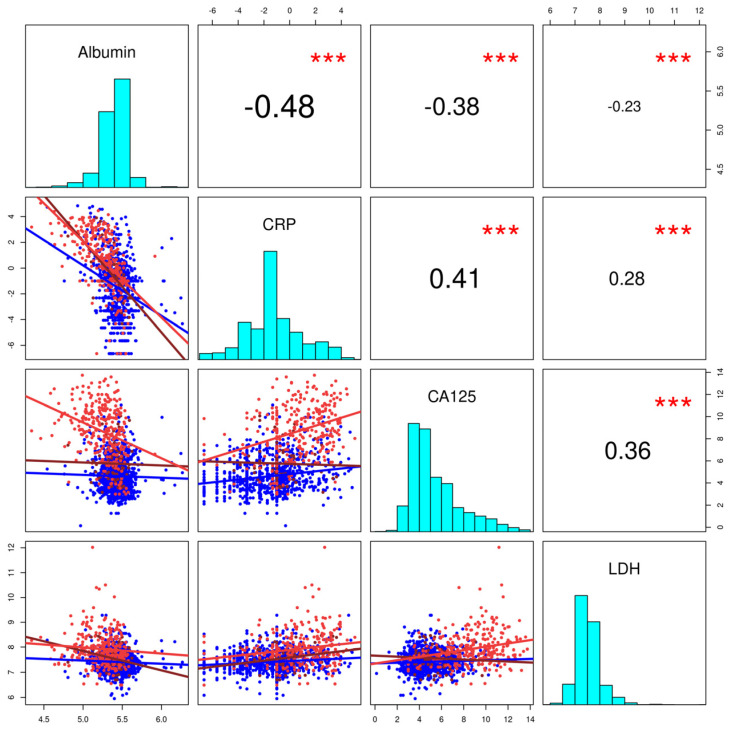

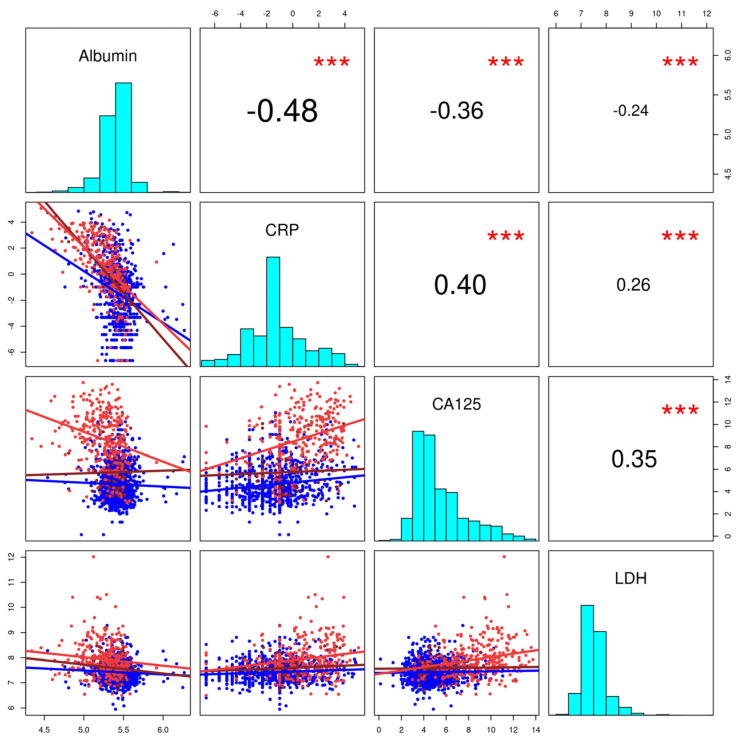
Correlation between albumin, CRP, CA125, and LDH (Log_2_ values) in two imputed datasets showing comparable results. Red dots, epithelial ovarian cancer (EOC); petrol dots, borderline tumor of the ovary; blue dots, benign; *** *p* < 0.001.

Correlations between the four most relevant markers included from the final model building process (see below; albumin, CRP, CA125, and LDH) were studied in two of the 20 imputed datasets (Figure 2, yielding one of the smallest models with four parameters; yielding one of the largest model with seven parameters). Overall, a significant moderate negative correlation was seen between albumin and CRP and significant weak negative correlations to both other markers (Figure 2). The highest correlation was consistently seen in the EOC group of patients (red color), but in the BOT group (dark red), a similar correlation was only seen between albumin and CRP and not with the other markers or the other markers with each other. 

### 3.3. Model Building

**Figure 3:**
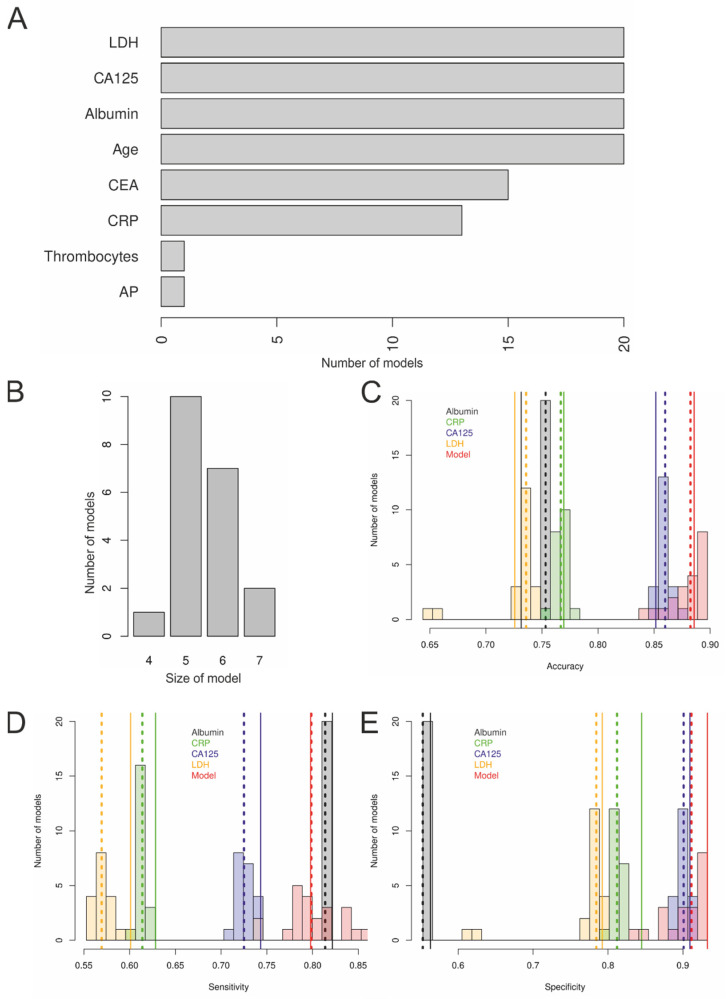
Building of the final models. (**A**) Four parameters were selected in all 20 (and the complete dataset) models, albumin, CA125, LDH, and age; (**B**) the models comprised four to seven parameters; (**C**) accuracy, (**D**) sensitivity, and (**E**) specificity of the most relevant single laboratory parameters compared to the 20 imputed models are shown, using a cut point optimized for the highest sum of sensitivity and specificity. The dotted lines represent the median values of the 20 imputed datasets. The solid line represents the value of the complete dataset.

Primarily, 20 imputed datasets were generated by multiple group wise imputations of missing values. Thereafter, all analyses were performed using the complete dataset and these 20 imputed datasets. The model building process using the relaxed lasso procedure (aiming at as sparse as possible models) with the complete dataset revealed a model with albumin, CA125, LDH, patients’ age, and thrombocyte count, and using the 20 imputed datasets revealed 20 models (one model comprising four, ten models comprising five, seven models six, and two models seven parameters; Figure 3B). The remaining parameters included in the final models were albumin, CA125, LDH, and patients’ age in all models, followed by CEA (*n* = 15), CRP (*n* = 13), thrombocyte count (*n* = 1 and the complete dataset), and AP (*n* = 1) in descending order. In Figure 3A, it is shown how often each singular parameter was included in the respective models, whereby four parameters were used in every model, i.e., age, LDH, CA125, and albumin. Calibration curves of all 20 lasso models were estimated and revealed to look very similar. Supplementary Figure S3 shows two models: one model with four parameters (A; one of the smallest models) and one model with seven parameters (B; one of the largest models). All models seemed to slightly underestimate the risk for malignancy, the larger model less so compared with the smaller model. The Brier scores for the four most relevant single parameters and the 20 models of the imputed datasets and the model of the complete dataset are shown in Supplementary Figure S4. The Brier scores (dependent on sample size) showed that CA125 was by far the best single predictor compared to albumin, CRP, and LDH, but also that the models comprising of four to seven parameters were slightly (mean 0.177 compared to mean 0.191 for CA125), but significantly (*p* = 1.9 × 10^−9^; *t*-test), better than CA125 alone.

Accuracies, sensitivities, and specificities with cut points optimized for the highest sum of sensitivity and specificity of the models compared to the most relevant single parameters are visualized in Figure 3C–E, respectively. The highest sensitivities were seen for albumin, the highest specificities were seen for CA125, and the highest accuracies were seen for the models.

Akaike information criterion (AIC) values for the 20 models from the imputed dataset were median −801.1 (IQR −813.4 −785.6; range −841.4 −763.0) and −312.3 for the model from the complete dataset. The small models (with four or five parameters) showed smaller AIC values compared to the larger ones (six to seven parameters).

**Figure 4:**
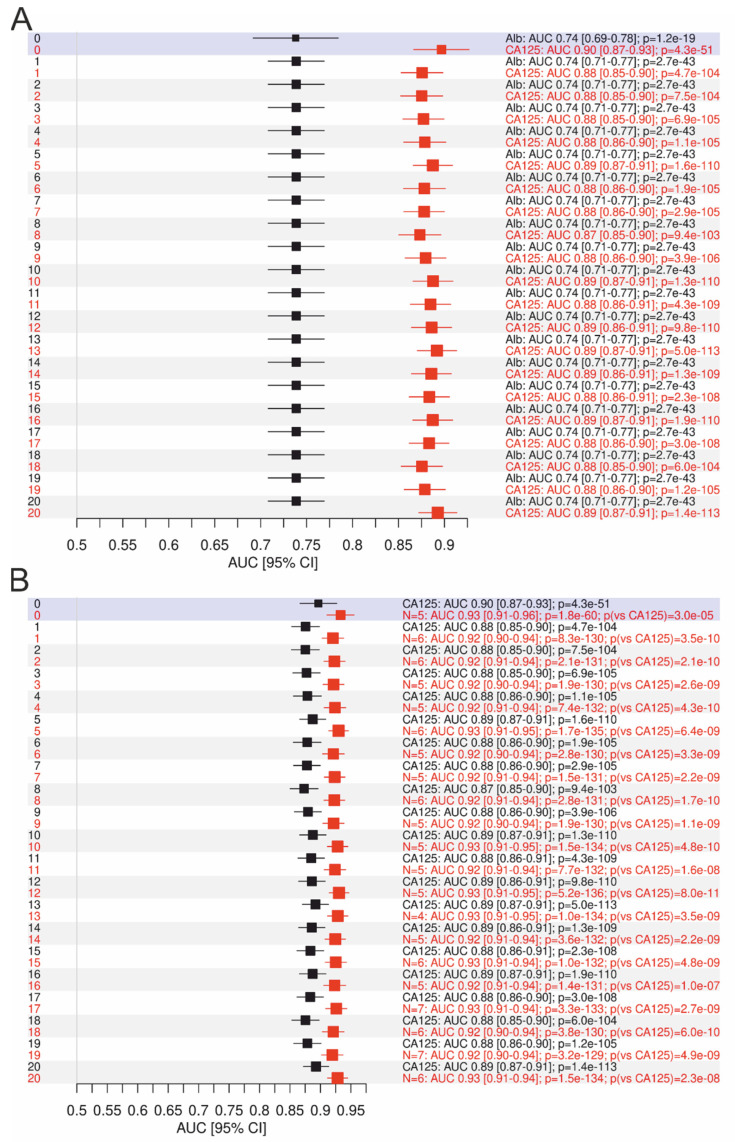
(**A**) The estimated AUCs (95% CI) of albumin (black) vs. CA125 (red) and (**B**) CA125 (black) vs. the models (red), performed with all 20 imputed datasets and the complete dataset (blue background). The first *p*-values compared the AUC estimates to 0.5 (as by chance). The last *p*-value (red) in (**B**) compared the CA125 AUC with the models AUC. *Y*-axis, number of imputed datasets; 0, the complete dataset.

In the next step, the AUCs with confidence intervals of albumin and the other single laboratory parameters (using all 20 imputed and the complete datasets) were estimated. Albumin with an AUC of 0.74 (95% CI 0.71–0.77) performed better than CRP or LDH, but not better than CA125 (Figure 4A and Supplementary Figure S5). Finally, the AUCs of the most relevant single parameter CA125 and the models were compared (Figure 4B), whereby all 20 lasso models were significantly better (AUCs 0.92–0.93; 95% CI 0.90–0.95) than CA125 alone (AUCs 0.88–0.89; 95% CI 0.85–0.91) and also the results from the complete dataset. 

**Figure 5:**
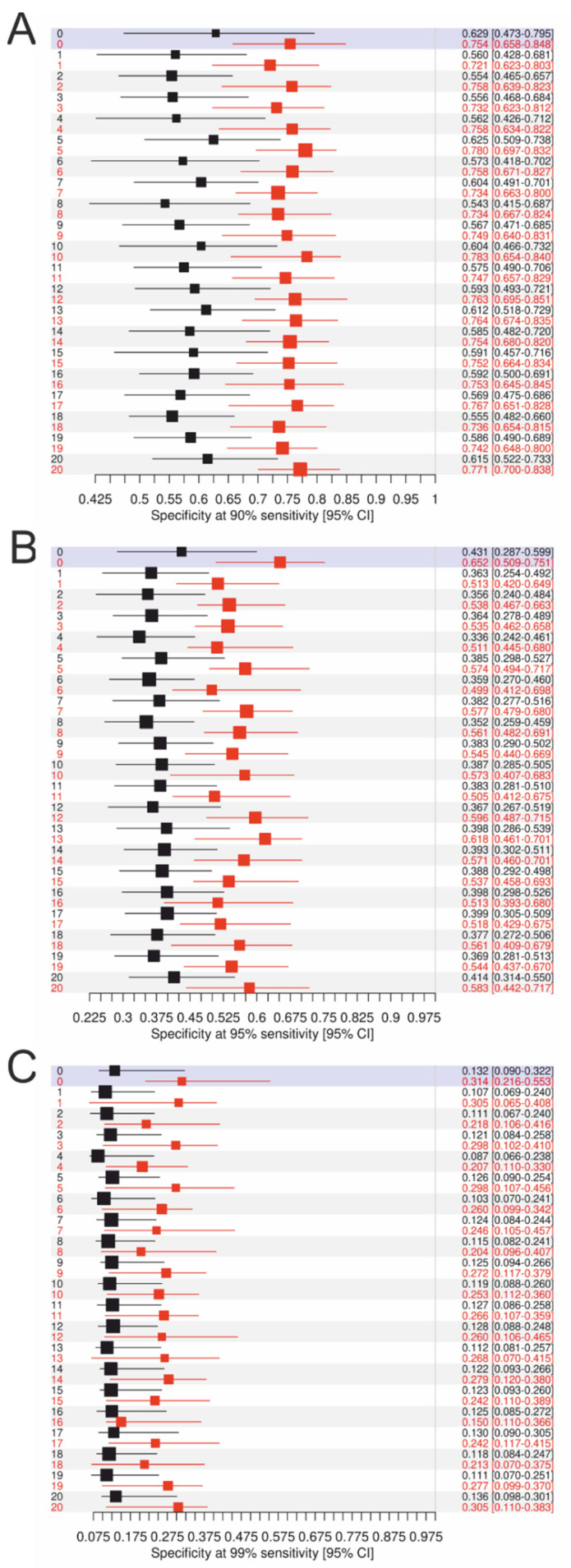
Specificities (95% CI) of CA125 (black) vs. the models (red) at a set sensitivity of (**A**) 90%, (**B**) 95%, and (**C**) 99%, respectively. *Y*-axis, number of imputed datasets; 0, the complete dataset.

In clinical routine, we need to correctly identify all patients with malignancy prior to surgery; thus, we aimed at reaching the highest sensitivities. Therefore, we set the sensitivity to the values 90%, 95%, and 99%, respectively, and estimated the corresponding specificities, comparing the strongest single parameter, CA125, and the models (Figure 5). We observed that at 90% sensitivity, the specificities of the models were approximately 76%, much better than CA125 showing specificities of only approximately 60%. At 95% sensitivity, specificities were already too low for a reliable multimarker diagnostic tool. 

Albumin was particularly sensitive and CA125 had a high specificity; the models comprising the combined parameters showed a better specificity compared to albumin alone. 

Using the standard clinical cut-off of 35 mg/dl for albumin, the sensitivity and specificity between benign/BOT and malign samples were 98.1% and 23.1%, respectively, and using the standard clinical cut off of 35 kU/L for CA125, the sensitivity and specificity between benign/BOT and malign samples were 25.3% and 15.3%, respectively. 

The median optimal cut offs of the four single diagnostic predictors over the 20 imputations and maximizing the sum of the specificities and the sensitivities were 40.26 mg/dl for albumin (no range, because this marker was not imputed), 84.80 (82.20–87.20; IQR 84.80–84.80) kU/L for CA125, 205.0 (182.0–205.0; IQR 203.8–205.0) U/L for LDH, and 0.80 (0.78–0.80; IQR 0.80–0.80) mg/dl for CRP. The corresponding sensitivities, specificities, and accuracies are shown in Figure 3C–E. 

**Figure 6:**
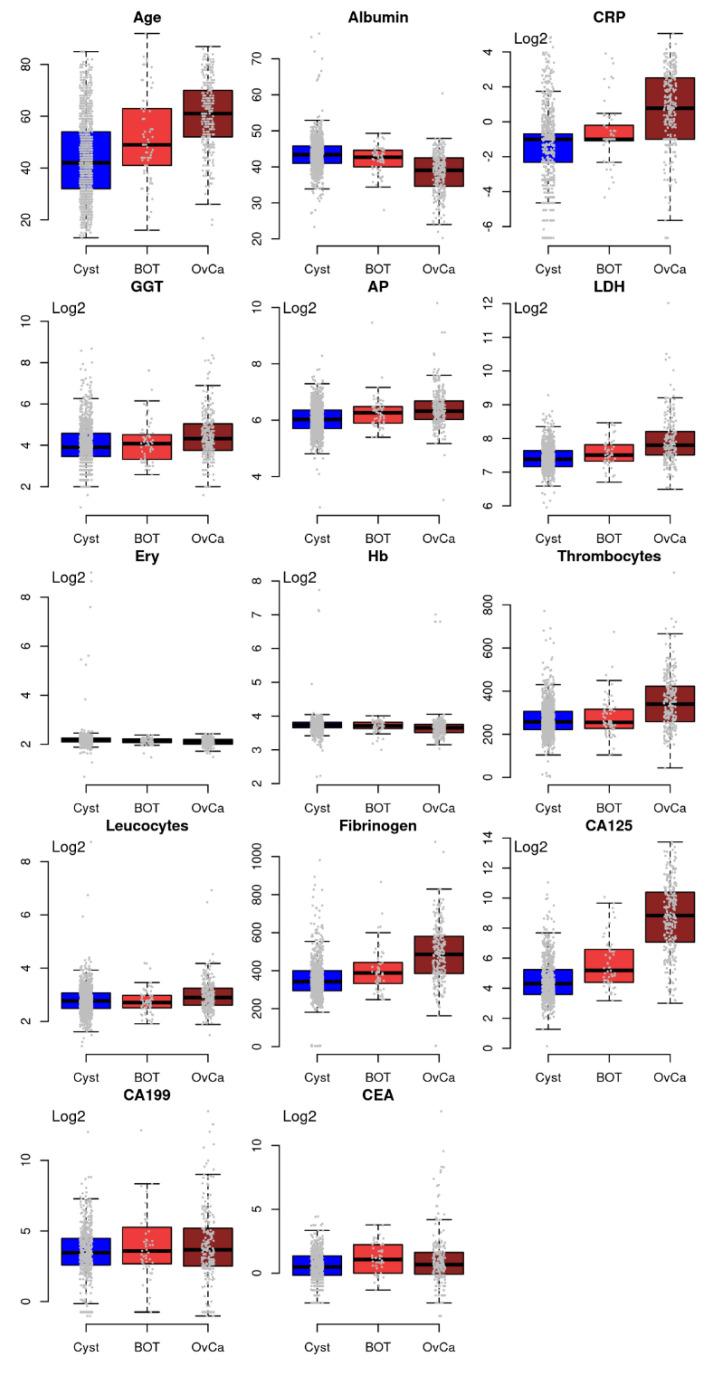
Characteristics of the single predictors compared between benign, BTO, and EOC. *Y*-axis: values are linear of log2 transformed, as indicated by the word “Log2”. Units of analytes are as provided in Table 1.

The abundant values in the three groups of the single predictors are shown in Figure 6.

### 3.4. Interactive Dynamic Nomogram

**Figure 7:**
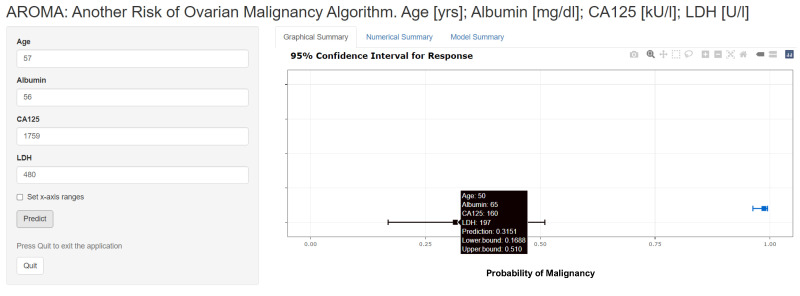
Interactive nomogram for predicting the malignancy of a suspect adnexal mass (https://pils.shinyapps.io/AROMA/) accessed on 21 June 2022. Two predictions are shown; the black one with a prediction of 31.5% malignancy and parameter values shown in the black box, and the blue one with a prediction of 98.7% malignancy and parameters shown in the left input box. The characteristics of the model can be seen following the “Model Summary” link. Used units are indicated in square brackets.

To provide a clinically usable model, all imputed datasets were averaged and used to build a model, including the four parameters, which were always selected during the relaxed lasso model building procedure, i.e., age, albumin, CA125, and LDH. This model is interactive and can be accessed here: https://pils.shinyapps.io/AROMA/ (accessed on 3 June 2022). Two typical cases are shown in Figure 7. The model summary can be accessed at the “Model Summary” link from inside the homepage. This model shows an AUC of 0.930 (95% CI 0.913–0.947) and specificities of 77.2%, 54.6%, and 19.0% for sensitivities of 90%, 95%, and 99%, respectively. Supplementary Figure S6 shows the ROC curves for the single predictor CA125, a model with albumin and CA125, and the four-parameter model used in the nomogram built with the averaged dataset, and compared the AUCs between albumin+CA125 and the four-parameter model.

## 4. Discussion

The current ESGO/ISUOG/IOTA/ESGE Consensus Statement on the preoperative diagnosis of ovarian tumors highlights that decision making should never be based only on one modality [3], and that besides adequate ultrasound expertise, accurate biomarkers are valuable in the treatment planning of patients with adnexal masses. Using laboratory parameters derived from a routine clinical setting, this study compared albumin to calculated models in 1552 patients undergoing surgery because of an adnexal mass. The final new models comprised four to seven parameters out of albumin, CA125, LDH, CRP, thrombocyte count, CEA, erythrocytes, AP, and patients’ age. 

Accurate biomarkers have been demonstrated to greatly improve the effectiveness of treatment and, thus, patient’s quality of life. The risk of ovarian malignancy algorithm (ROMA) comprising CA-125, HE-4, and menopausal status was investigated in a variety of clinical studies [5,16,17], but has not yet found its way into standard clinical use. Still, meta-analyses showed its superiority compared to single markers, such as CA-125 or HE-4 alone [18]. Carreras-Dieguez et al. only recently compared the role of the Copenhagen-index (CPH-I) and ROMA for the preoperative assessment of adnexal tumors in a large sample of more than 1000 patients, showing a consistent sensitivity and specificity (ROMA: 91.1% and 84.6%; CPH-I: 91.1% with 79.2%, respectively) with other reported studies [16,18,19]. Generally, the comparison of the sensitivity and specificity of biomarkers and probability indexes with the literature remains difficult, as the compared groups are often different (in some cases, BTO is included; in some cases, only EOC is included) and the selected cut-off points differ.

Although albumin showed a high sensitivity in the preoperative differentiation between benign and nonbenign adnexal masses, we confirmed that combined models resulted in a higher accuracy (AUC 0.88–0.89; 95% CI 0.85–0.91) compared to the use of single markers. Of note, we could also demonstrate that optimally dichotomized albumin had a higher sensitivity and specificity compared to dichotomization at the standard upper limit of normal. The following cut-off value was calculated to best dichotomize albumin: 40.26 mg/dl. Still, in the model building process, only continuous values were used to enhance reproducibility [20]. As albumin was particularly sensitive and CA125 had the highest specificity, the final calculated models all comprised both parameters and, thus, showed a better specificity compared to albumin alone. We compared specificities of the models at set sensitivities of 90%, 95%, and 99%. We observed that at 90% sensitivity, the specificities of the models were only at approximately 76%, still better than CA125 as a single parameter showing specificities of only approximately 60%. At 95% sensitivity, specificities were already too low, even worse at 99%. 

We primarily focused on albumin, as hypoalbuminemia is often diagnosed in cancer and associated with poor outcome [9,11]. In cancer patients, an increased catabolism and following cachexia seem to cause hypoalbuminemia [21]. Moreover, increased vascular permeability leads to a shift of albumin from the intravascular sector towards the interstitium, resulting in decreased serum albumin levels [22]. Besides the classic marker CA125 as the most utilized laboratory marker in the diagnostic workup of suspicious adnexal masses [5], we also looked at CRP, as its production during carcinogenesis indicates tumor cell growth, an immune-mediated host-defense reaction against malignant cells, or both [23,24]. Moreover, a correlation with tumor biology, i.e., reflecting tumor load and tumor aggressiveness [25], as well as the association of elevated serum levels and an increased risk for the presence of EOC/BTO were shown [12]. Additionally, LDH was selected in all final models. LDH was measured in patients with suspected organ damage or dysfunction, such as myocardial infarction, as it is released in the case of cell damage. Elevated LDH levels are associated with poorer prognosis in a variety of cancer types [26], and also seem to be useful in the discrimination of adnexal masses [14]. An elevated platelet count is frequently seen in cancer patients compared to healthy controls [27], and this finding could be confirmed in our study.

The good performance of the final combined models may be explained by the following reasons: first, patient selection. Our population included patients with suspicious adnexal masses resulting in an EOC/BTO prevalence of 31.2%. This fact strongly affected the test performance of the included biomarkers. Second, patient number. This study included 1552 consecutive patients with adnexal masses, thus, more than other biomarker studies [5,16,28,29]. Third, we included both EOC and BTO as the primary endpoints. We merged these two groups, as the type of surgery is similar and included in contrast to surgery for a benign adnexal mass: the maximum effort to prevent spillage, thorough an exploration of the whole abdomen, and in most centers, the mandatory presence of a gynecologic oncologist.

A limiting factor was the retrospective study’s design, although all patients were collected in a consecutive manner and all patients scheduled for surgery due to an adnexal mass were included in this study to avoid selection bias. Due to the retrospective character of the study, only laboratory parameters presented in our institutional standard preoperative work-up could be included in the study. Thus, HE-4 was not available for this analysis and multiple imputations had to be performed for missing values.

By developing combined scores, we were able to improve the accuracy compared to selected single parameters in the preoperative differentiation between benign and nonbenign adnexal masses. The included parameters were chosen due to their good performance. Additionally, in other already available predicting scores, the combination of parameters improved the discrimination of adnexal masses [30,31]. Compared to a variety of biomarker studies [12,13,14,32], these new models are applicable in a routine setting, as only routinely available parameters were included in the model building process. Besides classic laboratory parameters, patient age was included as a clinical parameter as to be found also in other classifiers such as the Copenhagen index (CA125, HE-4, and age) [31]. 

## 5. Conclusions

In the search for a more precise preoperative classification of adnexal masses, models using routinely available parameters and patients’ age showed a higher accuracy compared to selected single parameters. A final interactive model was provided, accessible at https://pils.shinyapps.io/AROMA/, (accessed on 21 June 2022) which could be used in a routine setting after respective validation. 

## Figures and Tables

**Table 1:** Patients’ characteristics dependent on final histopathological result.

	Benign (1192)	BTO (66)	EOC (294)	*q*-Value ^1^
Age (years), median (IQR)	42.0 (32.0–54.0)	49 (40.5–63.75)	61.0 (52.0–70.0)	1.04 × 10^−55^
GGT (U/L), median (IQR)	15 (11–24)	17 (10–24)	20 (13–33)	1.17 × 10^−07^
AP (U/L), median (IQR)	65 (52–82)	77 (59–90)	80 (65–103)	2.91 × 10^−18^
LDH (U/L), median (IQR)	167 (143–199)	182 (160–225)	223 (181–298)	7.31 × 10^−44^
Hemoglobin (g/L), median (IQR)	13.3 (12.6–14.1)	13.1 (12.4–14.1)	12.6 (11.4–13.5)	1.15 × 10^−11^
Platelets (G/L), median (IQR)	257 (222–306)	255 (226–318)	340 (258–423)	4.26 × 10^−29^
Leucocytes (G/L), median (IQR)	6.8 (5.6–8.4)	5.7 (4.6–7.9)	7.4 (6.1–9.5)	1.26 × 10^−05^
Fibrinogen (mg/dL), median (IQR)	324 (294–401)	388 (331–448)	486 (384–581)	3.20 × 10^−46^
CA19-9 (U/mL), median (IQR)	11.0 (5.9–22.3)	12.0 (6.1–46.7)	12.7 (5.7–37.4)	0.112
CEA (ng/mL), median (IQR)	1.4 (0.9–2.6)	2.1 (1.0–4.7)	1.6 (0.9–3.1)	6.52 × 10^−07^
CRP (mg/dL), median (IQR)	0.5 (0.2–0.6)	0.5 (0.5–1.0)	1.7 (0.5–5.7)	3.47 × 10^−47^
CA125 (kU/L), median (IQR)	19.8 (12.0–37.7)	36.0 (20.75–102.1)	455.9 (130.5–1389.0)	1.44 × 10^−114^
Albumin (mg/dL), median (IQR)	43.4 (41.0–45.8)	42.7 (39.9–44.7)	39.1 (34.6–42.5)	4.90 × 10^−46^

^1^ Kruskal–Wallis test corrected for multiple testing according to Bonferroni–Holm. BTO, borderline tumor of the ovary; EOC, epithelial ovarian cancer.

## Data Availability

The data presented in this study are available on request from the corresponding author.

## Supplementary Materials

The following supporting information can be downloaded at: https://www.mdpi.com/article/10.3390/cancers14133210/s1, Figure S1: Missing values; Figure S2: Quantile-quantile (Q-Q) plots of log_2_ transformed data of all parameters, Figure S3: Calibration curves of a model with four parameters (A) and one from a model with seven parameters (B), Figure S4: Boxplot of Brier Scores of the four most important single diagnostic markers, Figure S5: The estimated AUCs (95% CI) of LDH vs. CRP, Figure S6: ROC curves and AUC estimates from a model comprising different markers.
