# Supplementary material for: Defining Models to Classify between Benign and Malignant Adnexal Masses Using Routine Laboratory Parameters
Source: Cancers (Basel). 2022 Jun 30;14(13):3210. doi: 10.3390/cancers14133210 (PMC9264825; doi:10.3390/cancers14133210)
Supplement: Supplementary file 1 [file cancers-14-03210-s001.zip › cancers-1668555-supplementary.pdf]

Supplement

Defining models to classify between benign and malignant adnexal masses using routine laboratory parameters

Elisabeth Reiser [1], Dietmar Pils [3], Christoph Grimm [2*], Ines Hoffmann [2], Stephan Polterauer [2], Marlene Kranawetter [2] and Stefanie Aust [2]

[1] Gynecological Endocrinology and Reproductive Medicine, Medical University of Innsbruck, Innsbruck, Austria. elisabeth.reiser@i-med.ac.at
[2] Department of Gynecology and Gynecologic Oncology, Comprehensive Cancer Center (CCC), Medical University of Vienna, Vienna, Austria.
[3] Division of Visceral Surgery, Department of General Surgery, Comprehensive Cancer Center (CCC), Medical University of Vienna, Vienna, Austria.
* Department of Gynecology and Gynecologic Oncology, Gynecologic Cancer Unit, Comprehensive Cancer Center (CCC), Medical University of Vienna, Vienna, Austria. christoph.grimm@meduniwien.ac.at

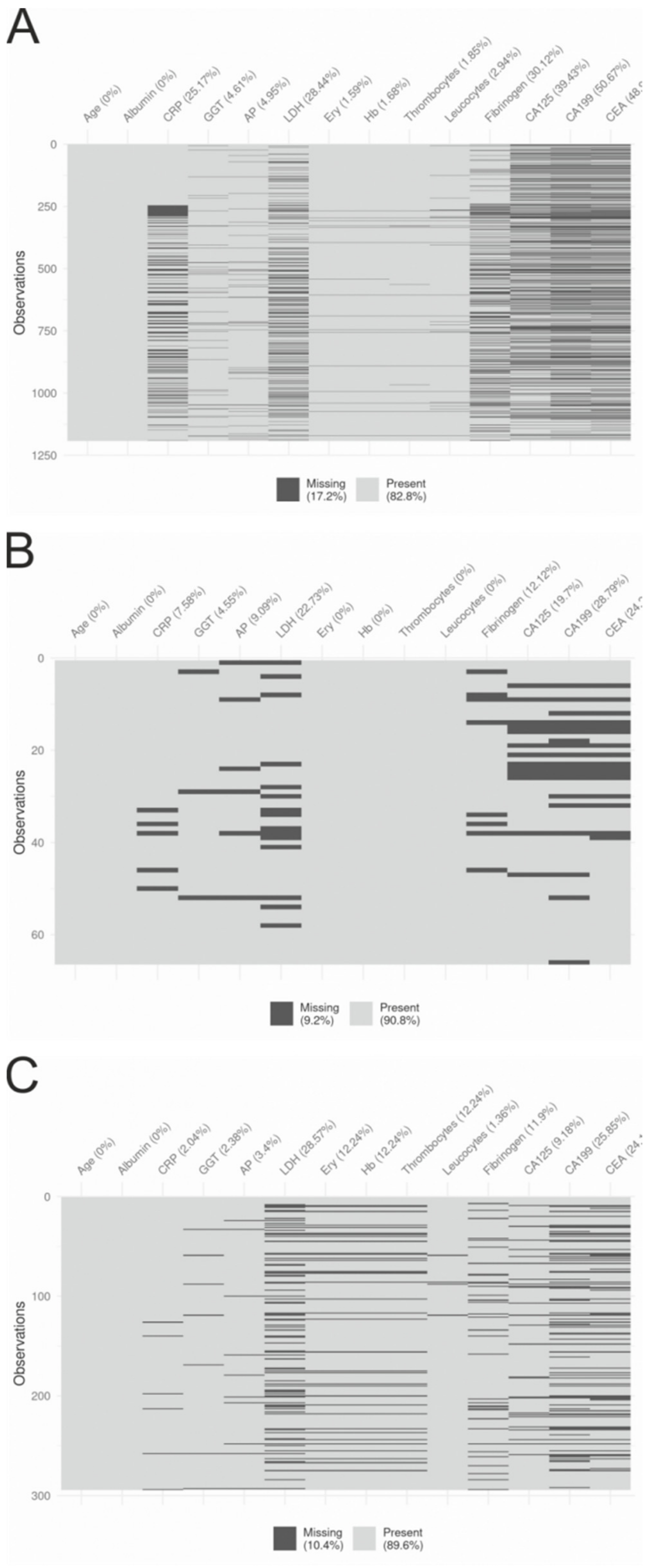

Suppl. Figure S1. Missing values, plotted with the vis_miss function from R-package naniar. Missing values were multiple (n=20) imputed in each group (benign, BTO, and malignant) by function mice of R-package mice using all available information including histology (which was not used for model building). Missing values are shown separately for patients with benign (A), borderline (B), or malignant disease (C).

A

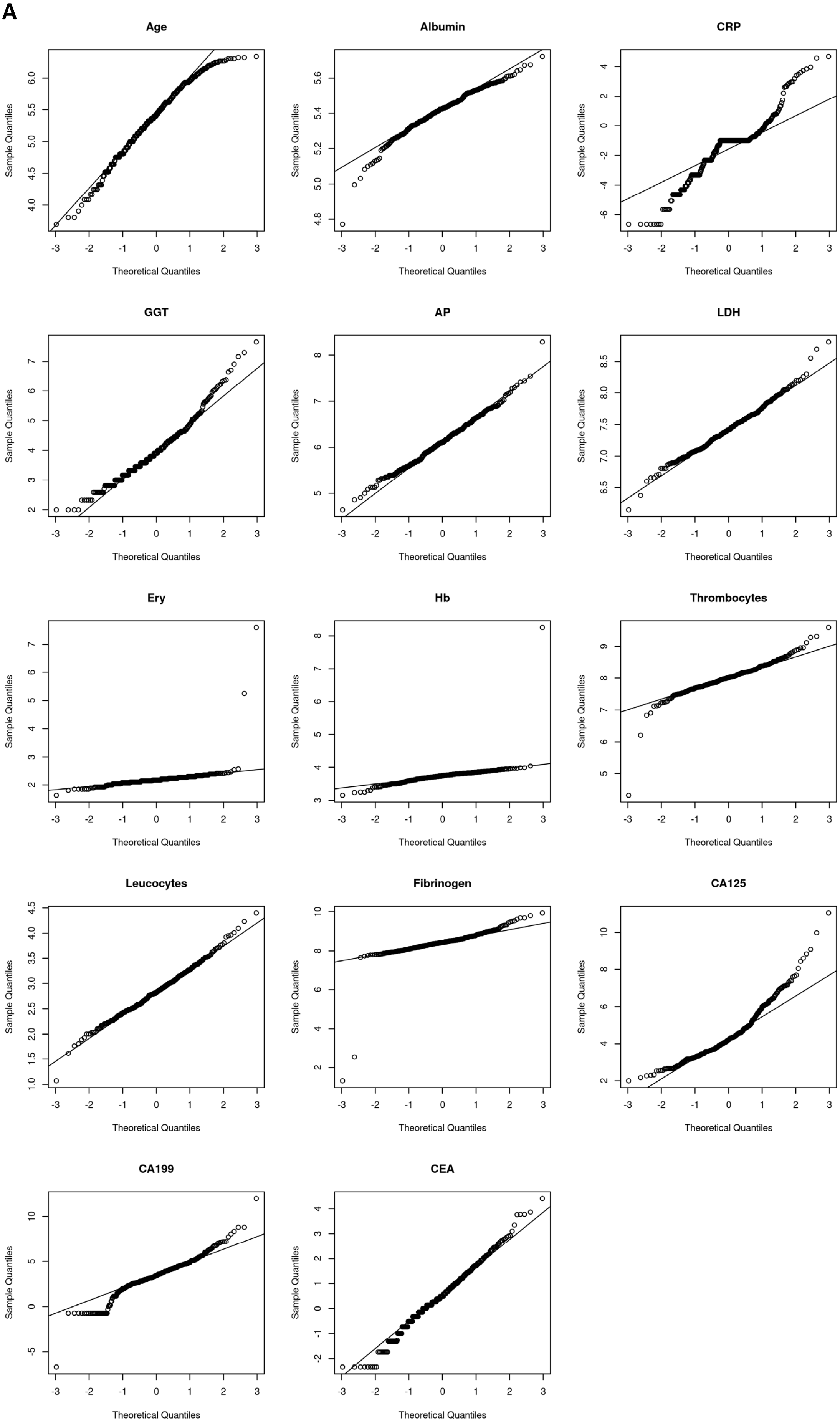
Age
Albumin
CRP
GGT
AP
LDH
Ery
Hb
Thrombocytes
Leucocytes
Fibrinogen
CA125
CA199
CEA
Sample Quantiles
Theoretical Quantiles

B

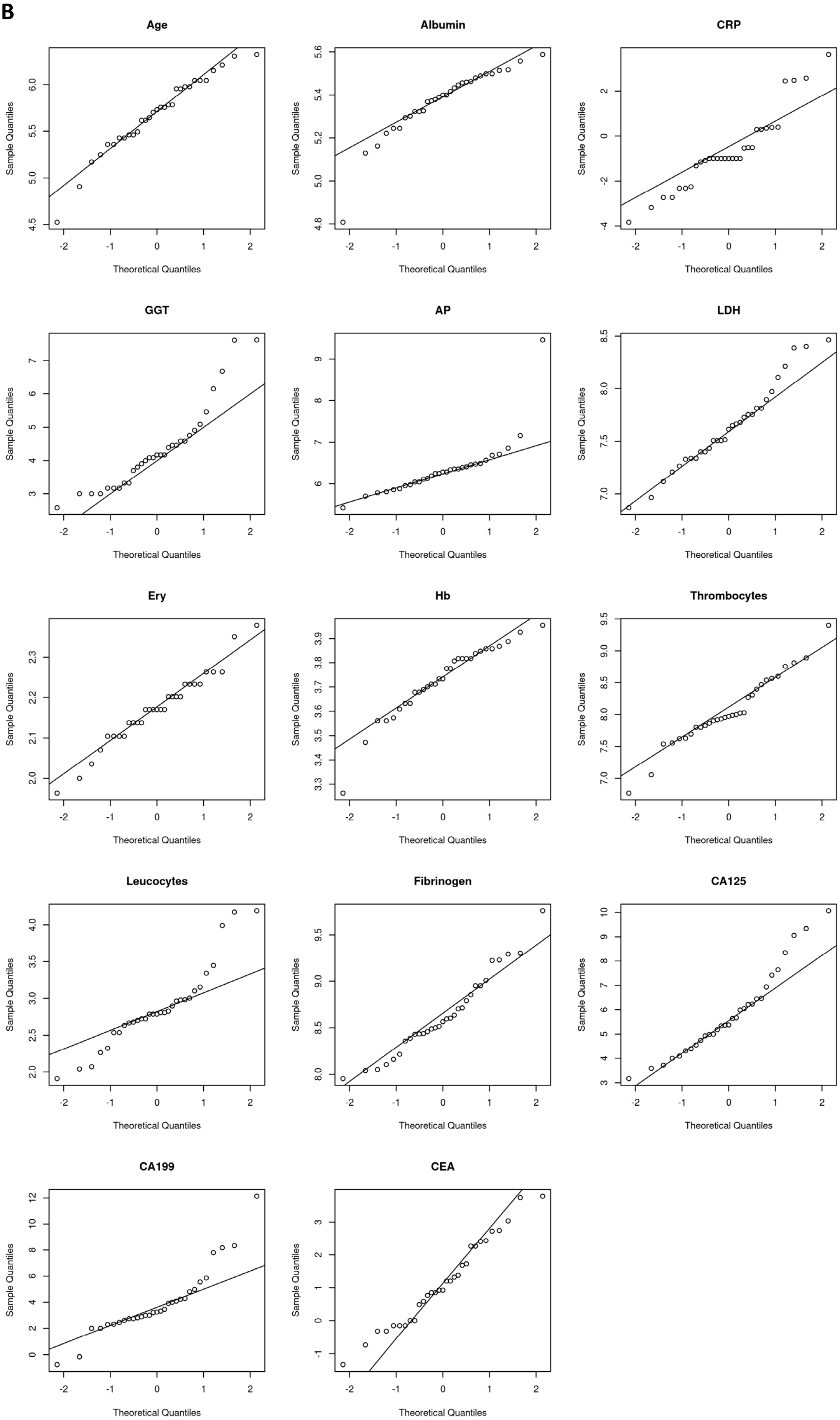
Age
Albumin
CRP
GGT
AP
LDH
Ery
Hb
Thrombocytes
Leucocytes
Fibrinogen
CA125
CA199
CEA
Sample Quantiles
Theoretical Quantiles

C

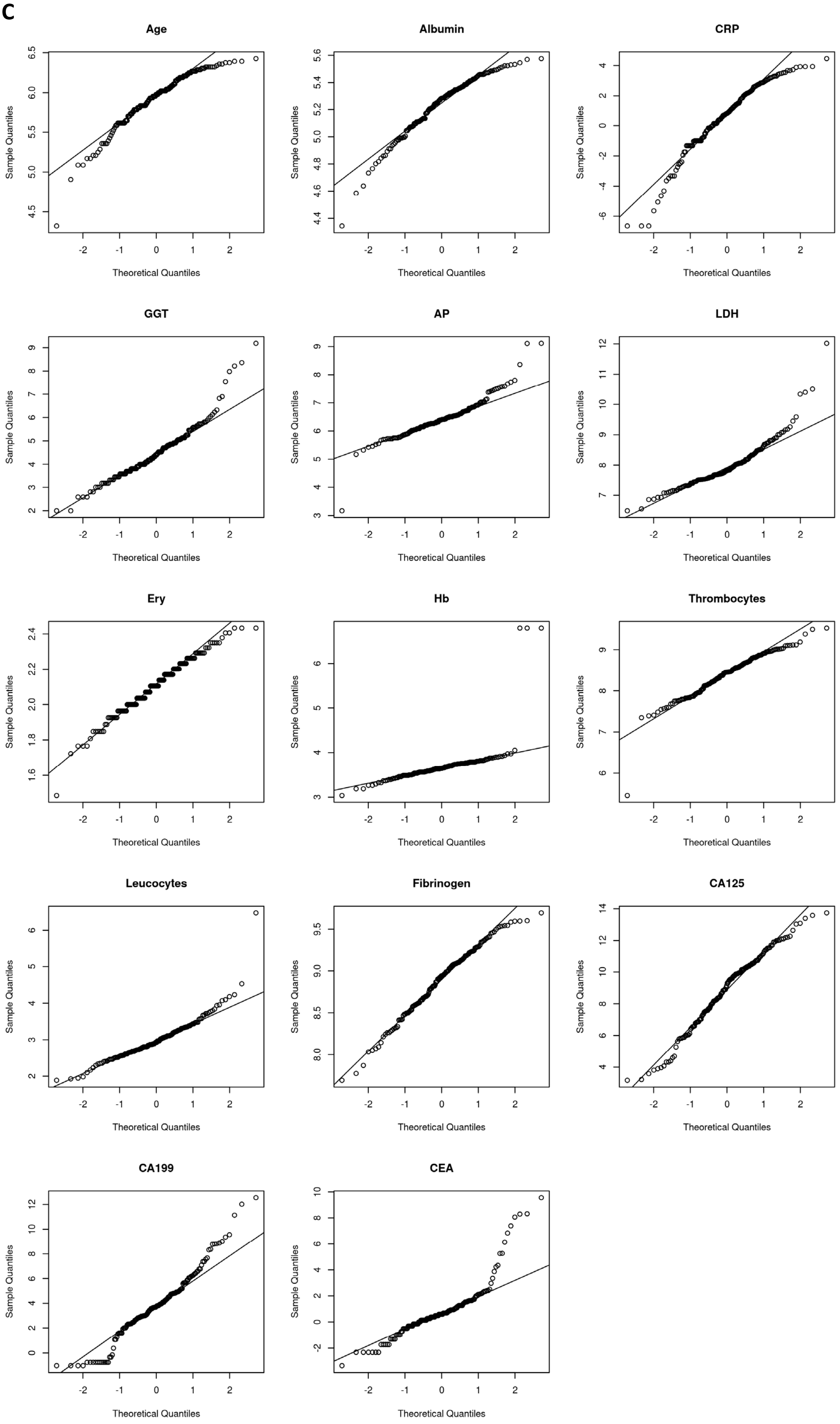
Age
Albumin
CRP
GGT
AP
LDH
Ery
Hb
Thrombocytes
Leucocytes
Fibrinogen
CA125
CA199
CEA
Sample Quantiles
Theoretical Quantiles

Suppl. Figure S2. Quantile-quantile (Q-Q) plots of log_2 transformed data of all parameters. (A) benign samples, (B) borderline samples, and (C) malignant samples.

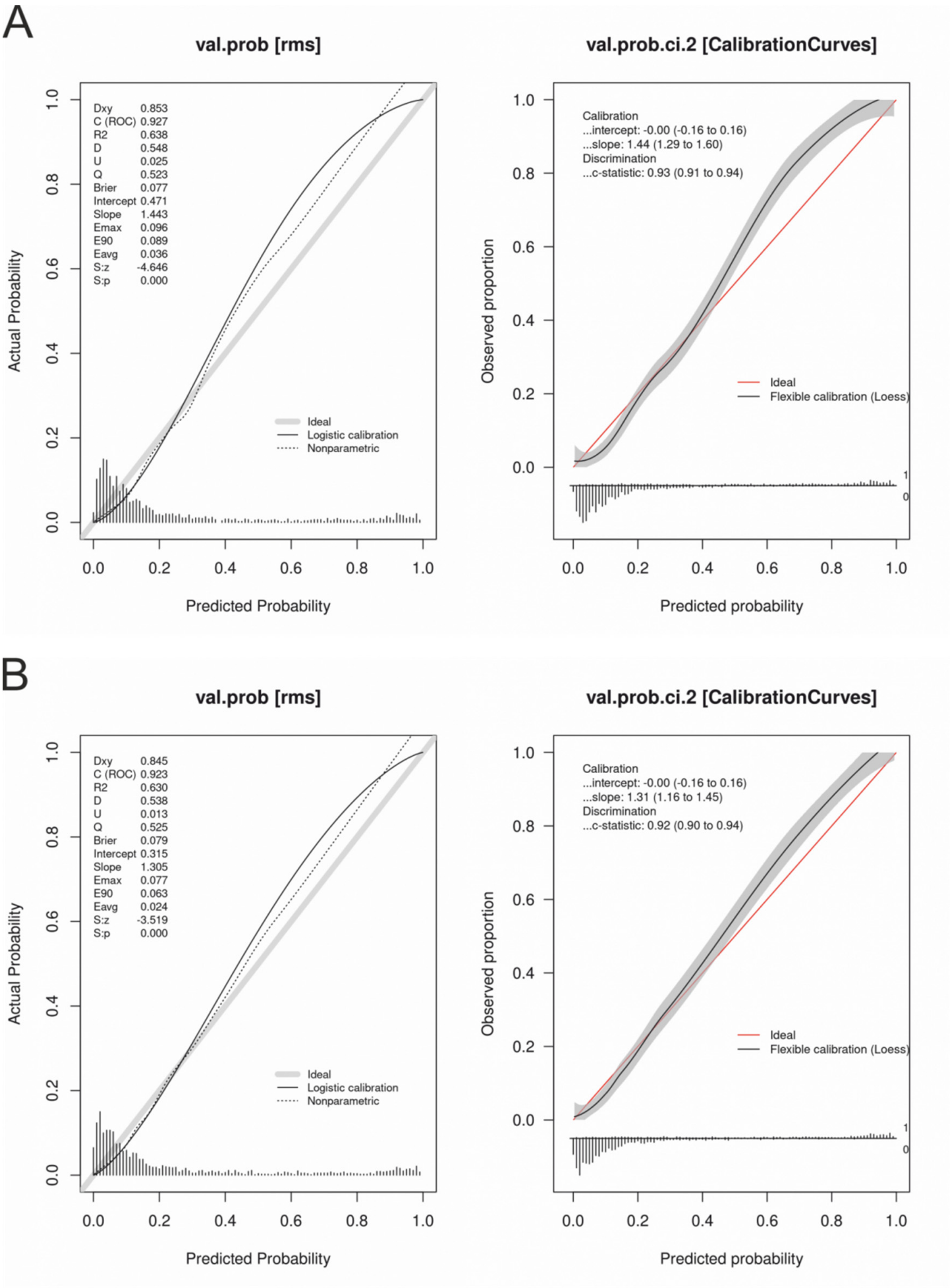

Suppl. Figure S3. Calibration curves of a model with four parameters (A) and one from a model with seven parameters (B). On the left side the plot derived from the val.prob function of the R-package rms 6.3-0 is shown (for the logistic calibration, a logistic regression using the logit of the probability as the predictor was fit and for the non-parametric curve, lowess smoother was used). On the right side a similar plot derived from the val.prob.ci.2 function from the R-package CalibrationCurves 0.1.2 is shown (using loess smoothing with pointwise 95% confidence intervals as grey band).

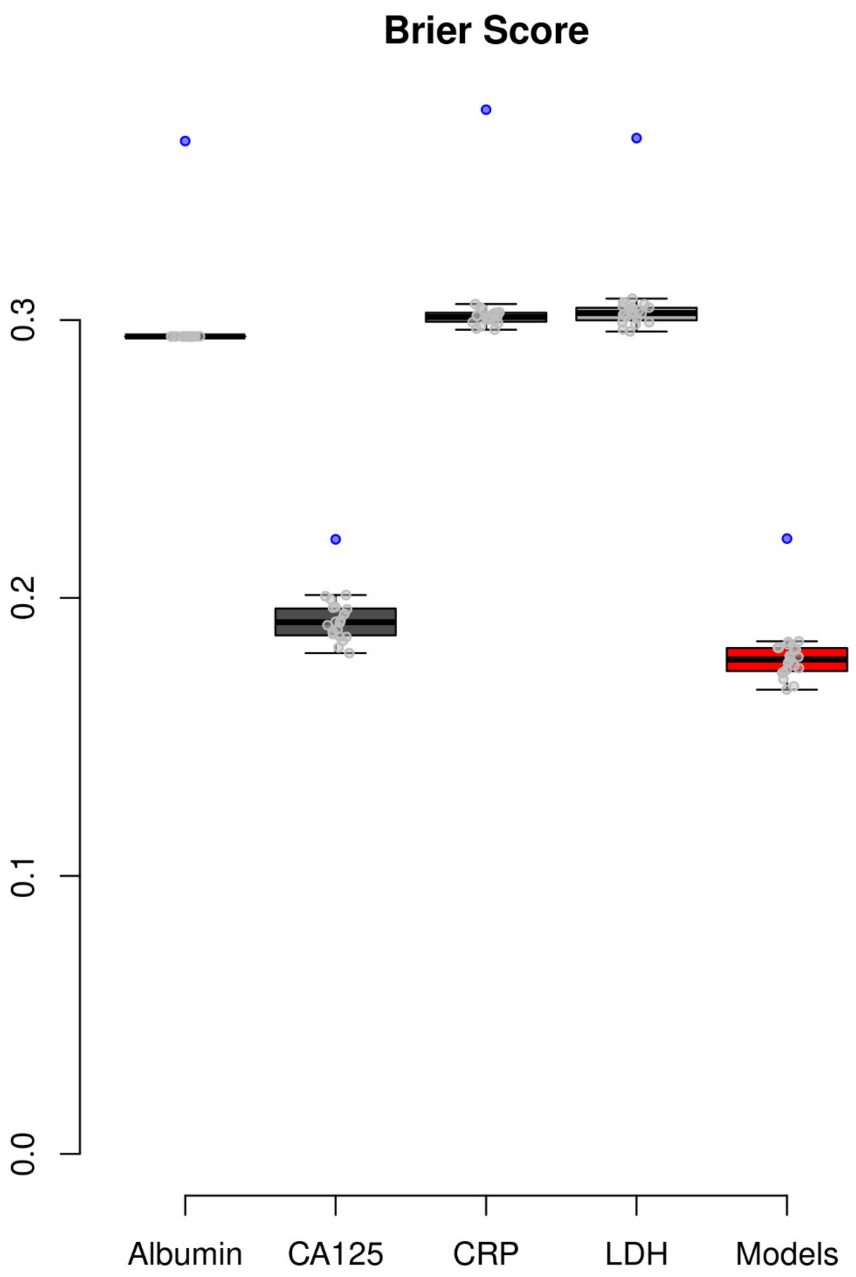

Suppl. Figure S4. Boxplot of Brier Scores of the four most important single diagnostic markers (grey colors) and the relaxed lasso-models (red). Shown are boxplots over all 20 multiple imputated datasets and in addition as blue dots from the complete dataset.

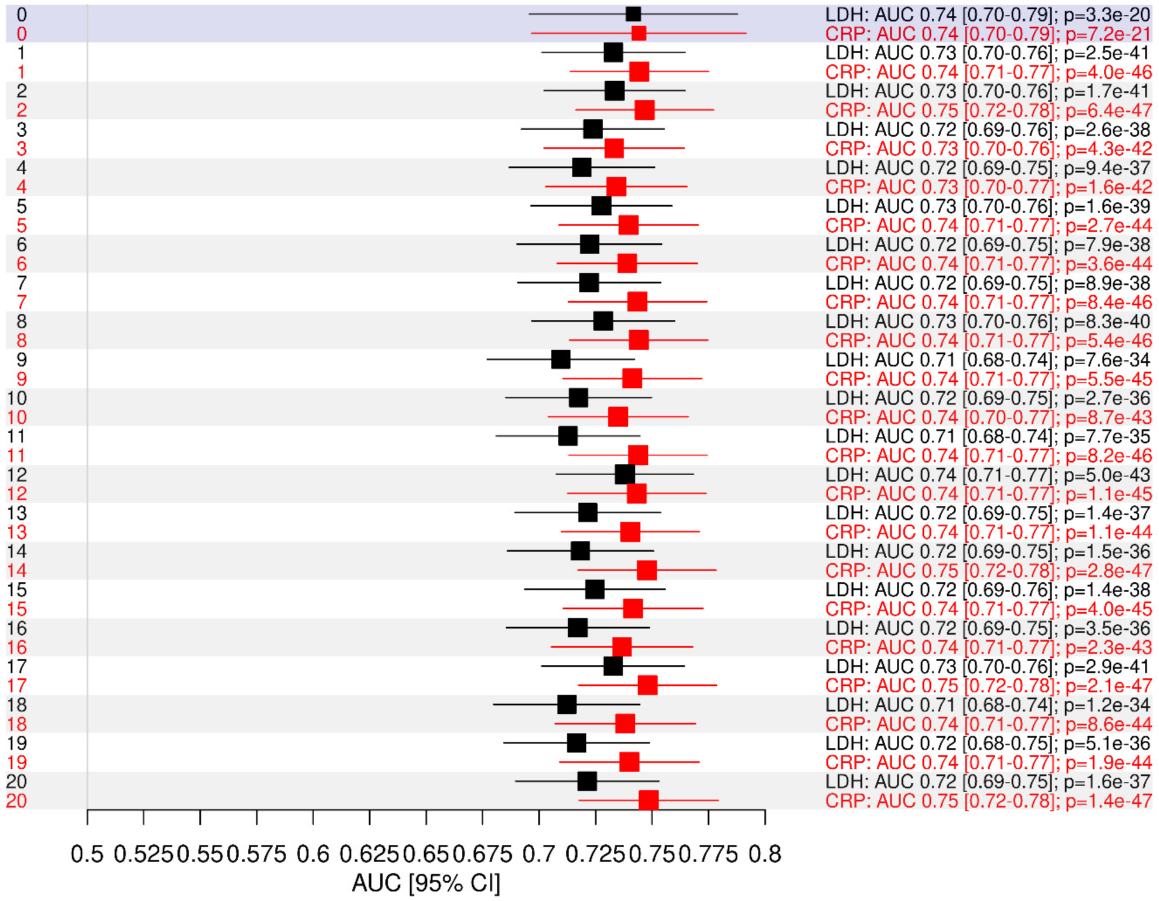

Suppl. Figure S5. (A) The estimated AUCs [95% CI] of LDH (black) vs. CRP (red); all 20 imputed datasets are shown (Y-axis, 1-20) and the complete dataset (0, blue background).

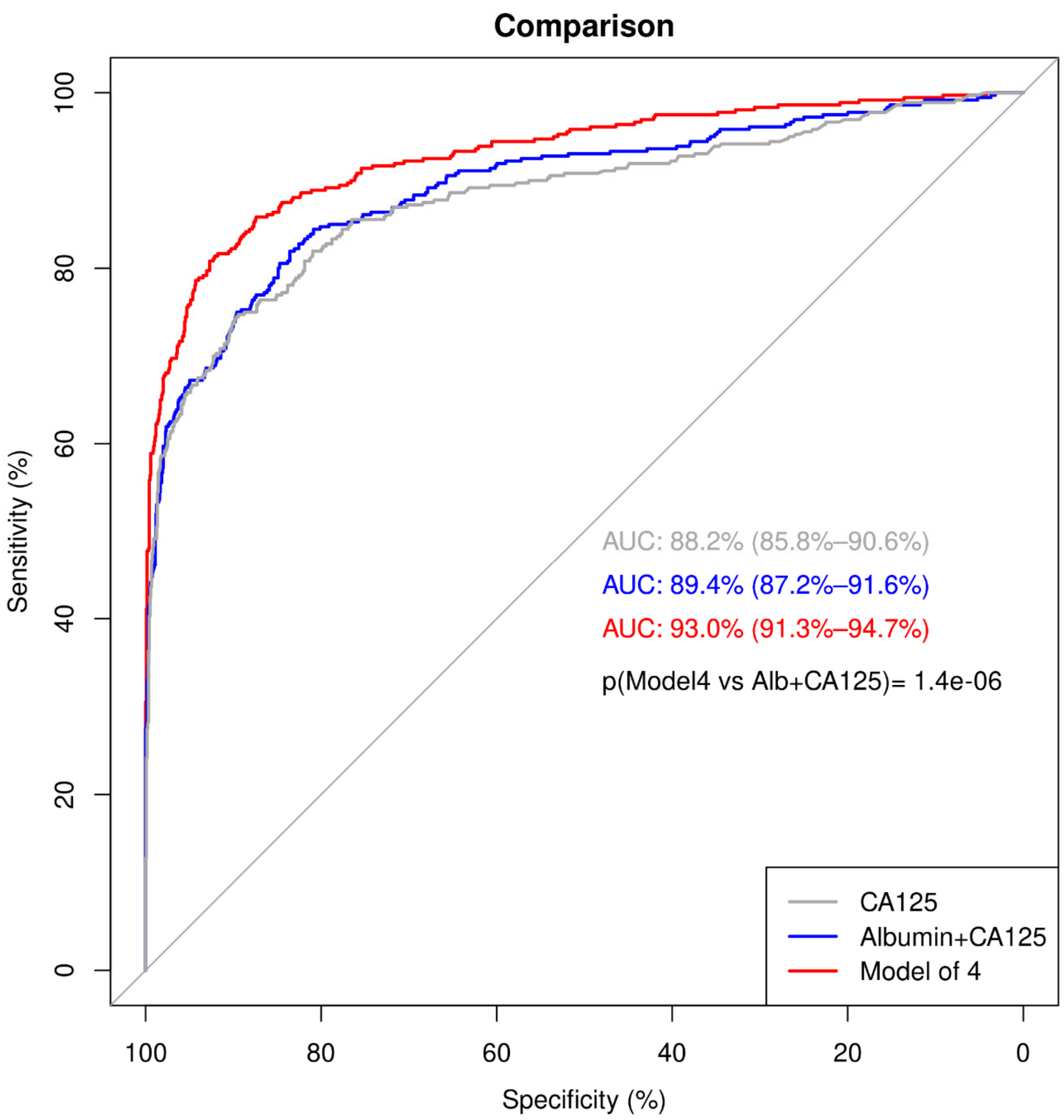

Suppl. Figure S6. ROC curves and AUC estimates from a model using the single parameter CA125 (grey), from a model comprised of albumin and CA125 (blue), and the four-parameter model (red, provided also as interactive dynamic nomogram, https://pils.shinyapps.io/AROMA/) using the averaged dataset. In black the p-value comparing the AUCs of the albumin+CA125 model with the four-parameter model is provided (calculated from 10,000 boostrap samples).
